# Post-Exposure JNJ-26366821 Treatment Attenuates Systemic Inflammation and Limits Cardiotoxic Risk

**DOI:** 10.3390/biom16091271

**Published:** 2026-09-02

**Authors:** Nabarun Chakraborty, Gregory P. Holmes-Hampton, Lily S. Neff, Vidya P. Kumar, Swapna Kannan, Amrita K. Cheema, Chandan Guha, Sanchita P. Ghosh, Rasha Hammamieh

**Affiliations:** 1 Integrative Systems Biology, Center for Military Psychiatry and Neuroscience, Walter Reed Army Institute of Research, Silver Spring, MD 20910, USA; nabarun.chakraborty2.civ@health.mil (N.C.); swapna.kannan2.ctr@health.mil (S.K.); 2 Armed Forces Radiobiology Research Institute, Uniformed Services University of the Health Sciences, Bethesda, MD 20889, USA; gregory.holmes-hampton@usuhs.edu (G.P.H.-H.); vidya.kumar.ctr@usuhs.edu (V.P.K.); 3 Department of Oncology, Lombardi Cancer Center, Georgetown University, Washington, DC 20057, USA; amrita.cheema@georgetown.edu; 4 Department of Radiation Oncology, Albert Einstein College of Medicine, Bronx, NY 10467, USA; cguha@montefiore.org

**Keywords:** acute radiation injury, thrombopoietin, heart, metabolites, lipids, proteins, therapeutic

## Abstract

Introduction: Thrombopoietin mimetics (TPOm) are known to increase blood cell counts, thereby ameliorating various diseases, including cardiomyopathy—a leading cause of mortality because of delayed radiotoxic effects. Previous preclinical studies demonstrated significant survival enhancement following lethal radiation exposure with a single dose of JNJ-26366821 in two mouse strains, CD2F1 and C57BL/6. Materials and Methods: In this study, a single dose of JNJ-26366821 (1.0 mg/kg) or saline was administered 24 h post-8.0 Gy total body irradiation (TBI) to male C57BL/6 mice. Mice were euthanized on days 1, 7, 15, and 30 post-TBI, and serum and heart samples were collected. Unirradiated mice treated with saline served as baseline controls. Inflammatory serum cytokines/chemokines, growth factors and multi-omics analyses, including proteomics, metabolomics, and lipidomics, were evaluated to assess therapeutic impacts. Results: JNJ-26366821 demonstrated overall positive effects, restraining radiation-induced inflammatory surges by 7 days post-TBI. Functional analysis revealed that JNJ-26366821 treatment in unirradiated mice activated cellular homeostasis, which persisted after post-exposure intervention in irradiated mice. The drug immediately reduced cell death, but networks supporting cardiovascular health, such as angiogenesis and vasculogenesis, remained inhibited 2 h post-intervention. By 7 days post-TBI, JNJ-26366821 mobilized molecules in heart tissue to reconstitute cellular development and maintenance. Protein synthesis and metabolism were activated between 15 and 30 days post-TBI, potentially compensating for radiation-induced muscle wasting. Conclusions: JNJ-26366821 demonstrated promising mitigating effects on cellular homeostasis and cardiovascular health when administered as a single dose at 24 h post-exposure.

## 1. Introduction

Radiation exposure, whether through intentional detonation of weapons or accidental exposure to radioactive materials, poses a significant global threat. This concern necessitates a deeper understanding of the biological effects of ionizing radiation and the development of robust medical countermeasures (MCM). Acute radiation syndrome (ARS) [1] encompasses the collective symptoms and health consequences following radiation exposure, with hematopoietic-ARS (H-ARS) being a specific sub-syndrome characterized by blood cell loss and anemia, and this particular clinical condition involves thrombocytopenia [2], which leads to increased bleeding risk, anemia, fatigue, and cardiovascular diseases [3].

Thrombopoietin (TPO) is the primary regulator of platelet production [4], binding to a cell surface receptor, namely c-Mpl, to activate signaling cascades that promote proliferation and differentiation of megakaryocyte progenitor cells, which subsequently increase the numbers of megakaryocyte and platelets. Thereby, TPO attenuates thrombocytopenia, leukopenia, and anemia [5]. In fact, TPO has been explored for treating conditions such as cardiac diseases [6], anemia and wound healing [7] which benefit from hematopoietic cell regeneration. Notably, Romiplostim [8,9,10], is the only FDA-approved TPO mimetic that promotes platelet recovery and survival in H-ARS [11].

The current study is poised to explore a novel TPO mimetic (TPOm), JNJ-26366821, as a potential radiation MCM [12,13]. JNJ-26366821 is a PEGylated peptide that embodies a 29 amino acid-long sequence doubly conjugated to two polyethylene glycol (PEG) structures [5]. Like Romiplostim, JNJ-26366821 shares no sequence homology with endogenous TPO; thus, the risk of auto-triggering antibody generation is minimal.

The translational potential of JNJ-26366821 is currently under evaluation. Studies probing the chronic toxicity, overall safety and tolerability of TPO mimetics in healthy volunteers have shown satisfactory results [14,15]. Like TPO, JNJ-26366821 also binds with c-Mpl receptors to activate the generation of hematopoietic stem cells and megakaryocytes [16,17].

Preclinical studies using two strains of mice demonstrated that a single subcutaneous administration of JNJ-26366821 (0.3 mg/kg or 1.0 mg/kg) 24 h post-TBI increased survival by approximately 30–90% in comparison to mice that received saline as a vehicle [12]. By attenuating thrombocytopenia and neutropenia, JNJ-26366821 accelerated the platelet and neutrophil recovery in irradiated CD2F1 mice, and confirmatory results were observed using the C57BL/6 H-ARS model [12].

These positive results with JNJ-26366821 for countering H-ARS and lethality served as the justification to probe its tissue-specific efficacy. JNJ-26366821 could be effective in treating certain long-term consequences of radiation, such as cardiovascular disorders. Notably, emerging data have identified thrombocytopenia as a potential risk factor for cardiovascular diseases [18]; consequently, TPO has been identified as a potential marker of cardiac diseases [6]. This knowledge is particularly important in radiobiology, since cardiovascular disease is a leading cause of radiation-induced late mortality [19,20]. Patients undergoing radiotherapy often suffer from long-term cardiotoxic complications such as pericardial disease, cardiomyopathy, fibrosis, coronary artery disease (CAD), and arrythmias [21,22,23,24,25,26,27]. The radiation-induced cardiovascular diseases, such as CAD, have an incidence rate of approximately 85%.

The objective of the current study was to evaluate the efficacy of JNJ-26366821 as a mitigator for radiation-induced heart damage in a preclinical model of H-ARS. C57BL/6 male mice were administered saline or JNJ-26366821 24 h after 8.0 Gy lethal TBI exposure. We analyzed the alterations in serum cytokines and heart proteomic, lipidomic and metabolomic profiles. A systems biology approach integrated this multi-domain data to understand the biological networks linked to irradiation pathophysiology in the heart and the ability of JNJ-26366821 to countermeasure the cardiac toxicity caused by lethal radiation.

## 2. Materials and Methods

### 2.1. Animals

The current study utilized male C57BL/6 mice aged 11–14 weeks obtained from The Jackson Laboratory (Bar Harbor, ME, USA) and housed in the Department of Laboratory Animal Resources (DLAR) at the Uniformed Services University of the Health Sciences (USUHS), an Association for Assessment and Accreditation of Laboratory Animal Care International (AAALAC) [16,28] accredited facility. Housing conditions were as follows: an automated 12 h light/dark cycle was used. Animals were fed Harlan Teklad rodent diet 8604 ad libitum and provided acidified water (pH 2.5–3.0) ad libitum. The temperature of the housing rooms was maintained at 21 ± 2 °C, and the relative humidity was maintained at 50 ± 10%. The housing rooms feature 10–15 cycles of fresh air per hour. The sample size for all experimental groups at individual timepoints are indicated (*n*) for each.

### 2.2. Ethics Statement

The animal study protocol (2017-04-006, expiration date: 23 April 2020) was approved by the Institutional Animal Care and Use Committee (IACUC) of the Armed Forces Radiobiology Research Institute using the principles and procedures outlined in the National Research Council’s Guide for the Care and Use of Laboratory Animals.

### 2.3. Drug Preparation

This study utilized a novel PEGylated TPO mimetic peptide, JNJ-26366821, developed by Janssen Research & Development, LLC (Raritan, NJ, USA) [15]. JNJ-26366821 was supplied to AFFRI in powder form. It was reconstituted in sterile saline (0.9% NaCl) to a 1.0 mg/kg concentration before use and protected from light during storage [12]. A single subcutaneous injection at the nape of the neck of saline or JNJ-26366821 was administered to mice 24 h after TBI.

### 2.4. Total Body Irradiation (TBI)

Mice were irradiated in the Armed Forces Radiobiology Research Institute (AFRRI) Cobalt-60 gamma irradiation facility as previously described [28]. Irradiations were conducted in well-ventilated plexiglass boxes (up to 8 mice per box) at a dose rate of ~0.6 Gy/minute (min) to total midline doses of 8.0 Gy. Dose rates were confirmed with an alanine/electron spin resonance (ESR) dosimetry system in the cores of acrylic phantoms found in all empty slots of the exposure rack in the plexiglass chambers. The alanine dosimeters were calibrated by the National Institute of Standards and Technology (NIST) in Gaithersburg, MD, USA, using their Cobalt-60 irradiator. Six sets of four alanine pellets were irradiated to six different doses: 0.975 Gy, 1.969 Gy, 5.05 Gy, 20.0 Gy, 50.0 Gy, and 100.0 Gy. These pellets were used to establish a calibration curve converting the electron spin resonance (ESR) radiation response in alanine to absorbed dose in water [29]. According to the NIST certificate, the dosimeters were irradiated surrounded by water-equivalent material in a calibrated cobalt-60 gamma-ray field at a dose rate of 60 Gy per hour. The irradiation temperature was maintained at 20 (±2) °C. The dose rate at the point of irradiation is expressed in terms of absorbed dose to water and is directly traceable to the NIST primary standard graphite calorimeter.

AFRRI Cobalt-60 Dose Mapping. Alanine dosimeters from the same batch as those from the calibration set were used to create a dose map for the AFRRI Cobalt-60 sources. The dosimeters were placed in five mouse phantoms in all empty slots of the exposure rack and irradiated to approximately 10 Gy. Actual doses in the mouse phantoms were determined using the NIST calibration curve [29]. Based on these doses and the irradiation time, the dose rates were calculated for each mouse location [12].

### 2.5. Housing and Care of Animals Following Irradiation

Following irradiation, mice were returned to their respective cages and monitored up to four times daily. The 10–20 day period beginning on day 8 was considered the critical period of peak mortality, which included additional checks of the animals and removal of moribund or deceased mice. Pre-determined criteria approved in the IACUC protocol were used to determine the moribund nature of the animals over the course of the study [12]. Mice that reached the criteria for early endpoints were euthanized with CO_2_ inhalation followed by cervical dislocation according to the guidance from the American Veterinary Medical Association.

### 2.6. Harvesting of Tissues for Molecular Assays

Irradiated mice (8 Gy) or non-irradiated mice (0 Gy) were randomized to experimental groups and administered a single dose of either JNJ-26366821 or saline subcutaneously at 24 h post irradiation. Mice were euthanized on days 1 (26 h post-radiation), 7, 15, and 30 (*n* = 5 per group) following blood collection through cardiac stick [12]. Serum was separated from whole blood in serum collection tubes (BD Microtainer tubes (Thermo Fisher Scientific, Waltham, MA, USA) and centrifuged at 2400× *g* for 10 min and used for cytokine estimation. After euthanasia, hearts were collected and processed for analysis.

### 2.7. Quantitative Estimation of Various Cytokines in Mouse Serum by Bio-Plex Assay

The Bio-Plex Pro Mouse Cytokine 23-plex assay kit (Catalog number: M60009RDPD, BioRad, Hercules, CA, USA) was used to analyze a panel of 23 inflammatory cytokines, chemokines, and growth factors using serum from different treatment groups as indicated above based on the vendor’s protocol [30]. Briefly, 50 µL of magnetic beads from the kit were combined with 50 μL of standards or serum samples diluted 1:4. The Bio-Plex Pro magnetic plate washer was used for all washing steps as per the manufacturer’s instructions. The biotinylated detection antibody cocktail and streptavidin-phycoerythrin were used as indicated in the manufacturer’s protcol. The plate was read on a Bioplex 200 (BioRad, Hercules, CA, USA) and the Bio-Plex Manager 6.1.1 was used for data acquisition.

### 2.8. Metabolomics Mass-Spectroscopy Assay

The metabolomics assay of heart samples was conducted as per the protocol described in the previously published work [28,31]. The QC data is shown in Figure S1. Targeted metabolomics was performed to quantify approximately 270 endogenous metabolites using a QTRAP^®^ 5500 LC–MS/MS system (SCIEX, Inc., Marlborough, MA, USA). All LC-MS grade reagents were bought from Fisher Scientific, Waltham, MA, USA. For tissue extraction, approximately 15–20 mg of frozen heart tissue was homogenized in 100 μL of extraction buffer (methanol/water, 50:50, *v*/*v*) containing 200 ng/mL debrisoquine (DBQ) as the internal standard for positive-ion mode and 200 ng/mL 4-nitrobenzoic acid as the internal standard for negative-ion mode. Tissue homogenates were vortexed for 30 s and incubated on ice for 20 min, followed by the addition of 100 μL chilled acetonitrile (ACN). Samples were then incubated at −20 °C for 20 min to facilitate protein precipitation and centrifuged at 13,000 rpm for 20 min at 4 °C. The resulting supernatants were transferred to LC–MS vials for analysis.

For chromatographic separation, 5 μL of each extract was injected onto a Kinetex column (2.6 μm, 100 Å, 100 × 2.1 mm; Phenomenex, CA, USA, Catalog number: 50-239-5919) using an SIL-30AC autosampler connected to an LC-30AD high-flow solvent delivery unit and a CBM-20A communication bus module (Shimadzu Scientific Instruments, Columbia, MD, USA). The LC system was coupled online to a QTRAP 5500 mass spectrometer (SCIEX, Inc., Marlborough, MA, USA) operated in both positive- and negative-ion modes. The mobile phases consisted of solvent A: water with 0.1% formic acid, and solvent B: acetonitrile with 0.1% formic acid. The flow rate was 0.2 mL/min. The gradient was initiated at 100% solvent A and held for 2.1 min, then decreased linearly to 5% solvent A over 12 min, held for 1 min, and finally returned to initial conditions (100% A) over 7 min for column re-equilibration. The autosampler temperature was maintained at 15 °C and the column oven at 30 °C.

Mass spectrometer source and gas settings were as follows: curtain gas 35 (arbitrary units), collision gas (CAD) set to medium, ion spray voltage +2500 V in positive mode and −4500 V in negative mode, source temperature 400 °C, nebulizer gas (GS1) 60, and heater gas (GS2) 70. Metabolites were monitored in multiple reaction monitoring (MRM) mode. Peak integration and quantification were performed using MultiQuant 3.0.3 (SCIEX, Inc., Marlborough, MA, USA). Peak areas were normalized to the corresponding internal standards. Data quality and reproducibility were ensured by (i) conditioning the LC column with pooled tissue quality-control (QC) extracts at the start of each batch, (ii) injecting pooled QC samples periodically (e.g., after every 10 study samples) to monitor shifts in signal intensity and retention time, and (iii) including solvent blanks between sets of samples to minimize carryover.

### 2.9. Targeted Lipidomics

Targeted lipidomics was performed to quantify 19 lipid classes, including diacylglycerols (DAG), cholesterol esters (CE), sphingomyelins (SM), phosphatidylcholines (PC), triacylglycerols (TAG), free fatty acids (FFA), ceramides (CER), dihydroceramides (DCER), hexosylceramides (HCER), lactosylceramides (LCER), phosphatidylethanolamines (PE), lysophosphatidylcholines (LPC), lysophosphatidylethanolamines (LPE), phosphatidic acids (PA), lysophosphatidic acids (LPA), phosphatidylinositols (PI), lysophosphatidylinositols (LPI), phosphatidylglycerols (PG), and phosphatidylserines (PS) using a QTRAP^®^ 5500 LC–MS/MS system (SCIEX, Inc., Marlborough, MA, USA).

For tissue lipid extraction, approximately 15–20 mg of frozen heart tissue was homogenized in 100 μL of chilled isopropanol containing class-specific internal standards using a homogenizer. Homogenates were vortexed for 1 min and kept on ice for 30 min, followed by incubation at −20 °C for 2 h to ensure complete protein precipitation and lipid extraction. Samples were then centrifuged at 13,000 rpm for 20 min at 4 °C, and the resulting supernatant was transferred to LC–MS vials for analysis.

For chromatographic separation, 5 μL of each extract was injected onto an XBridge amide column (3.5 μm, 4.6 × 100 mm; Waters) using an SIL-30AC autosampler connected to an LC-30AD high-flow solvent delivery unit and CBM-20A communication bus module (Shimadzu, MD). The LC system was interfaced online with a QTRAP 5500 mass spectrometer (SCIEX, Inc., Marlborough, MA, USA) operated in both positive- and negative-ion modes. The mobile phases consisted of solvent A: acetonitrile/water (95:5, *v*/*v*) with 10 mM ammonium acetate, and solvent B: acetonitrile/water (50:50, *v*/*v*) with 10 mM ammonium acetate. The flow rate was 0.7 mL/min. The gradient was initiated at 100% solvent A, changed to 99.9% A over 3 min, then to 94% A over the next 3 min, and to 25% A over the subsequent 4 min, followed by a wash at 100% solvent B for 6 min and re-equilibration to 100% solvent A over 6 min. The autosampler temperature was maintained at 15 °C and the column oven at 35 °C.

Mass spectrometer source parameters were as follows: curtain gas 30 (arbitrary units), CAD set to medium, ion spray voltage +5.5 kV in positive mode and −4.5 kV in negative mode, source temperature 550 °C, nebulizer gas (GS1) 50, and heater gas (GS2) 60. Lipid species were monitored in MRM mode and quantified relative to their respective internal standards.

Peak integration and quantification were performed using MultiQuant 3.0.3 (Sciex). Peak areas were normalized to the corresponding internal standard for each lipid class. Data quality and reproducibility were assessed by (i) conditioning the LC column with pooled QC samples at the start of the run, (ii) injecting pooled QC samples periodically throughout the batch to monitor shifts in signal intensity and retention time, (iii) analyzing NIST reference plasma prepared in parallel to assess instrumental variance, and (iv) including solvent blanks between sets of samples to minimize carryover. Following peak integration and normalization to internal standards, raw metabolite/lipid intensities were further normalized across samples using Probabilistic Quotient Normalization (PQN) to correct for sample-to-sample differences in overall dilution/concentration, including variability introduced by differences in individual tissue sample input mass. Briefly, a reference spectrum was calculated as the median intensity profile across all quality-control (QC) samples, and a sample-specific dilution coefficient was derived from the median of the quotients between each sample’s metabolite/lipid intensities and the corresponding reference intensities. Each sample’s data was then divided by this coefficient prior to downstream statistical analysis.

### 2.10. Sample Preparation for Proteomic Analysis

Approximately 25–30 mg of tissue was homogenized for 30 s in 400 µL of 100 mM ammonium bicarbonate (containing 1% sodium dodecyl sulfate) on an ice bath followed by vortexing for an additional 30 s. Tissue suspensions were centrifuged at 4 °C for 10 min at 16,000× *g*. A volume of 100 µL of supernatant was transferred to a 2.0 mL tube containing 900 µL of pre-chilled acetone and incubated at −20 °C overnight. After overnight incubation, samples were centrifuged at 4 °C for 10 min at 8000× *g*. The acetone was decanted without disturbing the white pellet. Pellets were washed with 450 µL of pre-chilled acetone, allowed to dry for 10 min, then reconstituted in 250 µL of 50 mM ammonium bicarbonate. A BCA assay was used to determine protein concentration (ThermoFisher Scientific, Rockville, MD, USA, Catalog number: 23227). The protein concentration in each sample was adjusted to 40 µg/200 µL in 50 mM ammonium bicarbonate. Samples were reduced with 5 mM Dithiothreitol (DTT) for 60 min at 56 °C and alkylated with 15 mM iodoacetamide for 30 min at 37 °C in dark conditions. The reaction was quenched with the addition of 5 mM DTT followed by overnight digestion with 2.5 ng/µL Trypsin Gold (Promega, Inc., Madison, WI, USA, Catalog number: V5280) at 37 °C. Trypsin was deactivated by heating for 10 min at 90 °C. Samples were cooled to room temperature and acidified to a pH of 3 using 0.1% trifluoroacetic acid (TFA) in H_2_O. The protein digests were purified on microspin C18 columns (The Nest Group Inc., Ipswich, MA, USA, Catalog number: HEM S18V) then eluted using 2 × 400 µL of 80% ACN/H_2_O acidified with 0.1% formic acid followed by 2 × 400 µL of 50% ACN/H_2_O acidified with 0.1% formic acid. The collected fractions were evaporated under vacuum and reconstituted for LC-MS/MS analysis.

### 2.11. Nano-Liquid Chromatography-Tandem Mass Spectrometry (Nano-LC-MS/MS)

Dried samples were reconstituted in 40 uL of 0.1% formic acid containing Pierce Peptide Retention Time Calibration Mixture (ThermoFisher, Rockville, MD, USA, Catalog number: 88321). A Thermo Electron Exploris 480 mass spectrometer system (Thermo Fisher Scientific, Inc., Waltham, MA, USA) with an Easy Spray ion source connected to a Thermo 75 μM × 15 cm C18 Easy Spray column was used. A sample volume of 2 μL was injected, and the peptides were eluted from the column by an acetonitrile and 0.1% formic acid gradient at a flow rate of 0.3 μL/min over 2 h. Precursor and fragment ions were acquired in the Orbitrap mass analyzer (Thermo Fisher Scientific Inc., Waltham, MA, USA) over an *m*/*z* range of 375–1500 with a resolution of 120,000. Precursor fragmentation was carried out using the higher-energy collisional dissociation (HCD) method utilizing normalized collision energy (NCE) of 30. The fragment ions were acquired at a resolution of 30,000. For internal mass calibration, the lock mass option was enabled with polysiloxane ion (*m*/*z*, 445.120025) from ambient air.

### 2.12. Proteome Analysis

All raw LC-MS/MS data was processed with Proteome Discoverer v2.4.1 (Thermo Fischer Scientific, Inc., Rockville, MD, USA) using the Sequest search engine (Thermo Fischer Scientific, Inc., Rockville, MD, USA) against the mouse UniProt database. A fragment ion mass tolerance of 0.020 Da and a parent ion tolerance of 15 PPM were used in the Sequest search. Carbamidomethyl of cysteine was specified in Sequest as a fixed modification. Oxidation of methionine was specified in Sequest as a variable modification. Scaffold Q+ v4.11.1 was used to validate MS/MS-based peptide and protein identifications. Peptide threshold was set to a probability of 99% or greater for acceptance. Protein threshold was set to FDR 1.0% or less and contained at least 2 identified peptides. Proteins that contained similar peptides and could not be differentiated based on MS/MS analysis alone were grouped to satisfy the principles of parsimony. Proteins sharing significant peptide evidence were grouped into clusters.

### 2.13. Statistical Analysis

All statistical analyses of the blood immune profile were performed using GraphPad, version 10 (Prism Inc., San Diego, CA, USA) with a significance threshold at *p* < 0.05. For the omics analysis, the Principal Component Analysis (PCA) tools R package 2.25.0 was used to run and plot a PCA of the samples with a 95% confidence level within each timepoint. All the samples were within the confidence interval, and there were no outliers detected. Principal Component 1 (PC1) and PC2 of each sample were extracted, plotted by bar and whisker plots, and 2-way ANOVA was conducted to understand the impacts of drug and time since irradiation on the PC scores. Subsequently, for each dataset (proteins, metabolites, and lipids), a two-way repeated measures ANOVA was performed to identify those features that were perturbed by time and treatment (Saline vs. JNJ-26366821) in the irradiated mice using the rstatix package; the significance threshold was at *p* < 0.1. Features that met this threshold at any given factor, namely time, treatment and time × treatment were curated for subsequent functional analysis. In parallel, a *t*-test comparative analysis was performed between the naïve mice with and without JNJ-26366821 treatment to capture the impact of the drug in naïve mice, and the significance threshold was at *p* < 0.05. A Venn analysis between these two sets of differentially expressed molecular features identified those that were perturbed only due to the cumulative impacts of time and drug in the irradiated mice. This screened list of statistically significant proteins, metabolites, and lipids were uploaded to Ingenuity Pathway Analysis (IPA, 2025 summer) software (QIAGEN, Inc., Germantown, MD, USA). Activation z-scores were calculated by IPA with a cutoff > |1.5| to curate significant pathways.

## 3. Results

**Figure 1:**
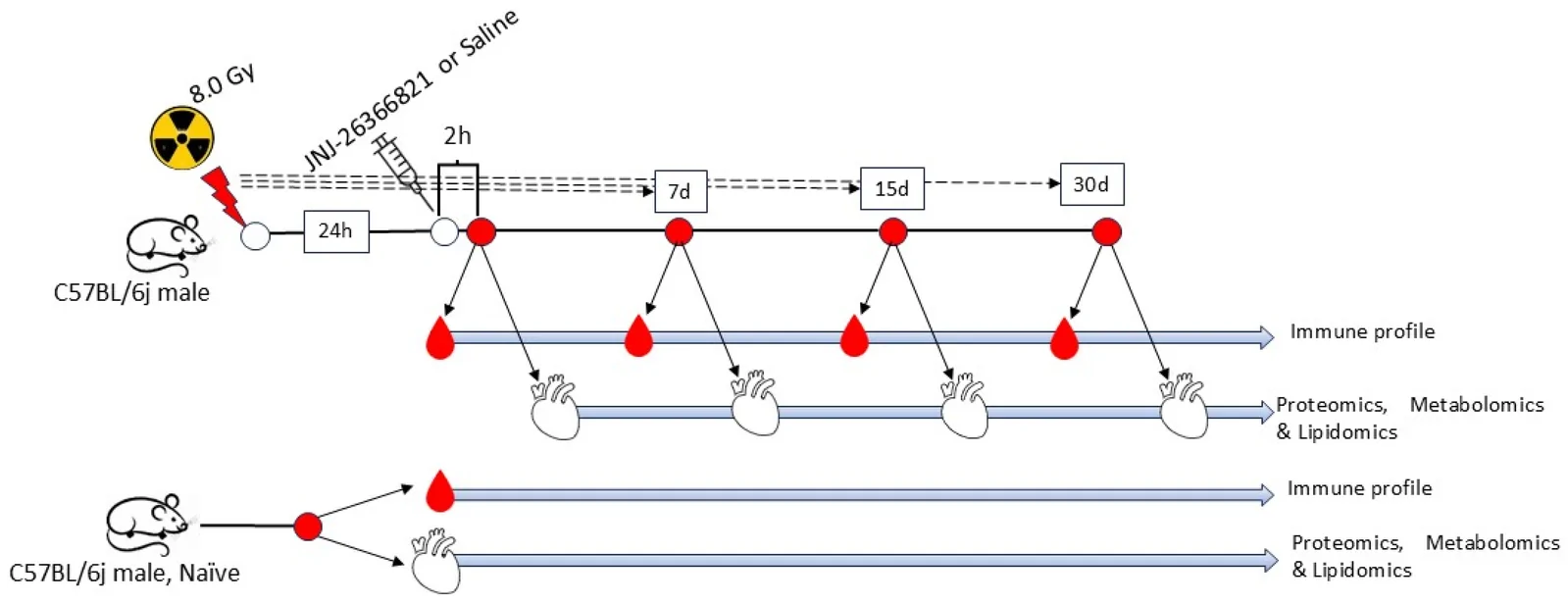
Experimental workflow of the research study showing the administration of the JNJ-26366821 drug 24 h after 8.0 Gy radiation exposure. A parallel cohort of naïve mice was euthanized as the baseline. The dashed arrows show the timeline and the red dots represent the time of euthanizing the mice and collecting heart and blood samples. The white dots represent the timepoints, when no euthanasia took place, but other procedures were conducted, such irradiation and drug administration. First round of euthanasia took place 2 h after drug administration.

Male C57BL/6 mice were randomized and received either 0 Gy or 8.0 Gy TBI. Twenty-four hours later, mice received one subcutaneous injection of JNJ-26366821 (1.0 mg/kg) or saline. Mice were euthanized on days 1, 7, 15, and 30 (1 d, 7 d, 15 d, and 30 d, respectively) post-TBI. The time gap between the JNJ-26366821 administration and the first batch of euthanasia was 2 h, and these samples were labeled as 1 d post-TBI samples. A naïve cohort was euthanized to represent the baseline of the study. Serum samples were collected post-mortem for cytokine analysis, and hearts were processed for targeted metabolomics, lipidomics, and proteomics analyses (Figure 1). For this study, non-irradiated animals predictably did not experience any mortality; among animals irradiated at 8 Gy, we observed ~17% mortality over the course of the study. Given the progression of H-ARS, animal mortalities were not observed at the early timepoints (Days 1 and 7) and all mortalities occurred at the later timepoints of collection (Days 15 and 30). This mortality rate is in line with our expected mortality rate for this dose of radiation.

### 3.1. JNJ-26366821 Administered 24 h After TBI Showed Positive Impact in Blood Immune Profile

**Figure 2:**
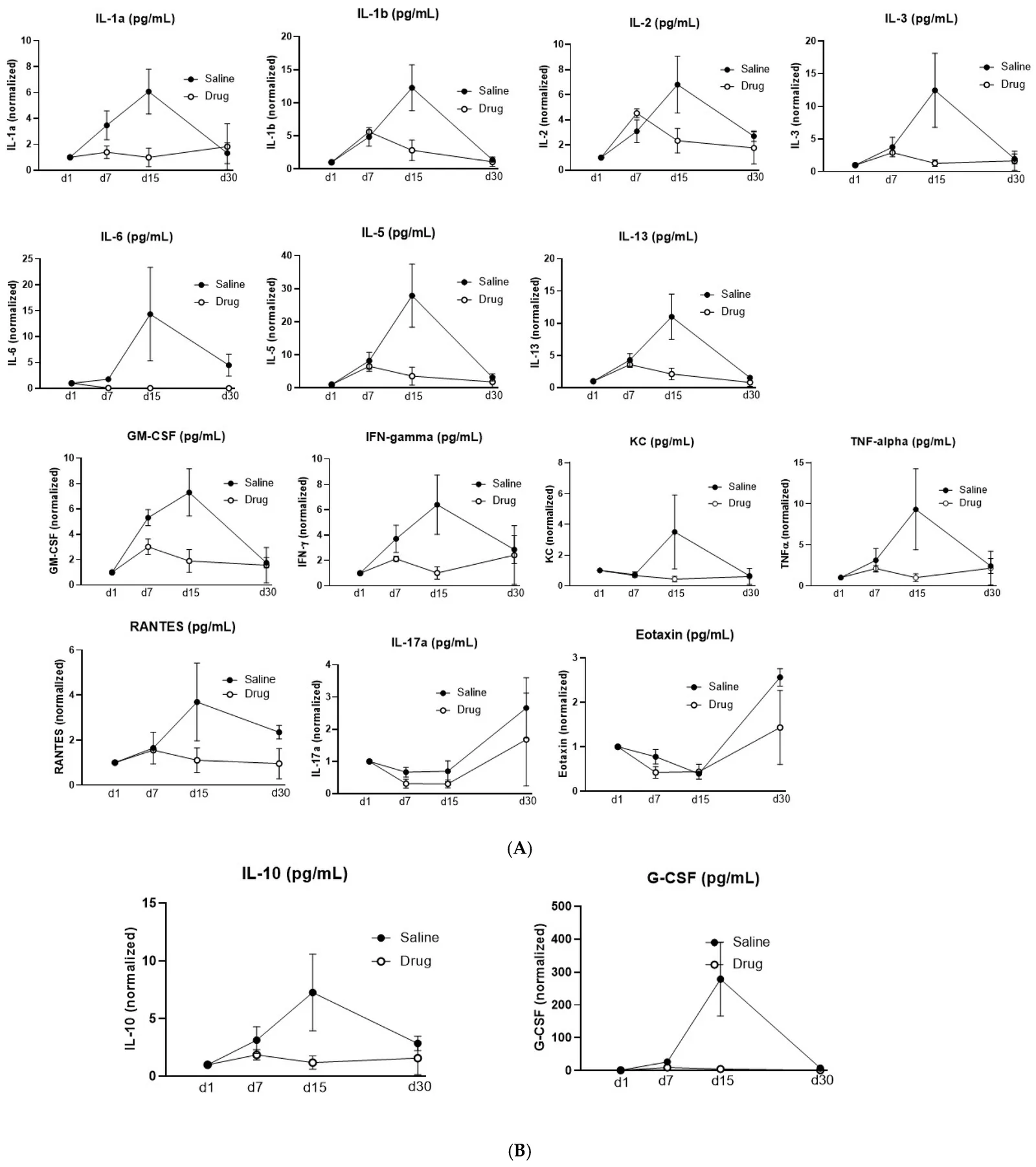

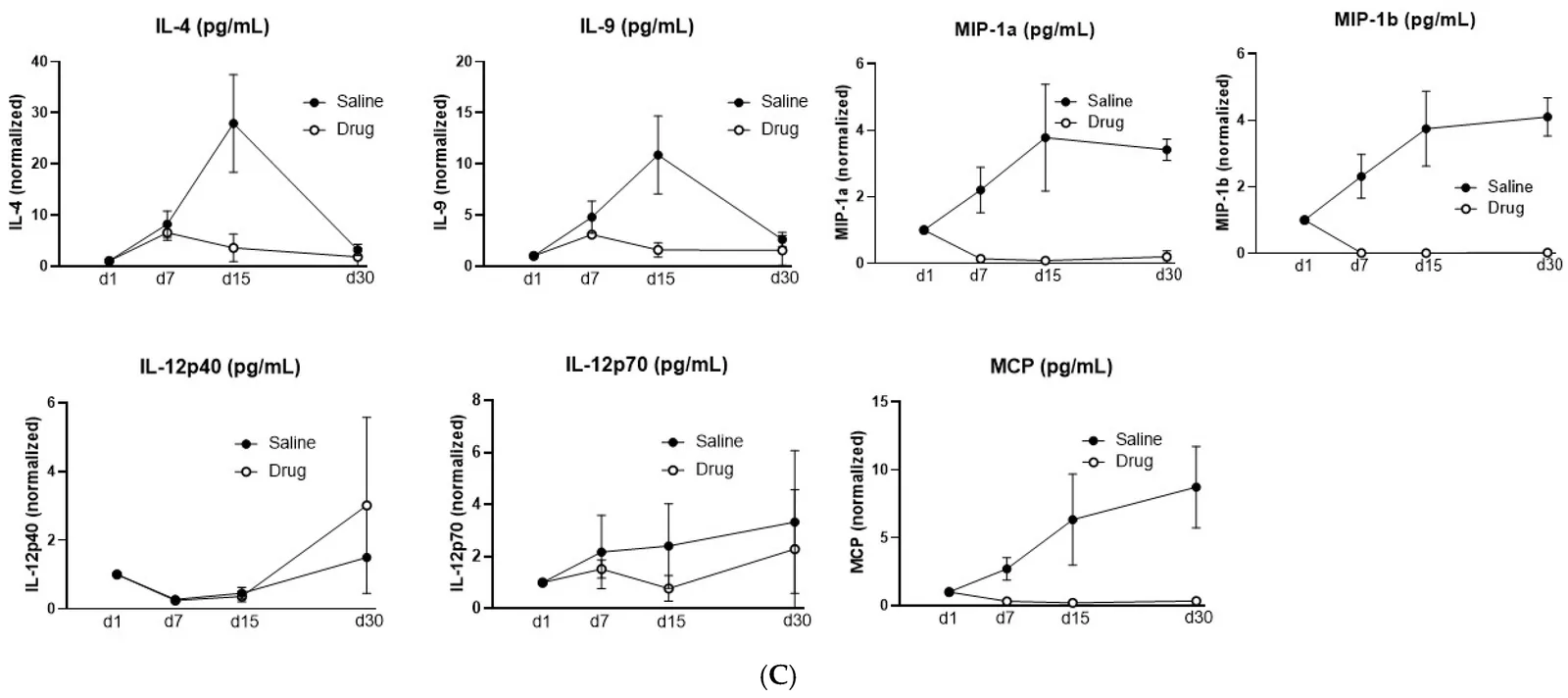
Longitudinal profile of a selected panel of inflammatory proteins in JNJ-26366821 and saline-treated mice. All data were normalized to day 1 samples. (**A**) Pro-inflammatory molecules (IL-1a, IL-1b, IL-2, IL-3, IL-5, IL-6, IL-13, GM-CSF, IFN-gamma, KC, TNF-alpha, RANTES, IL-17a, and Eotaxin) (**B**) Anti-inflammatory molecules (IL-10 and G-CSF) and (**C**) molecules related to immune functions and pre both pro- and anti-inflammatory (IL-4, IL-9, MIP-1a, MCP, MIP-1b, IL-12p40, and IL-12p70).

To understand the role of JNJ-26366821 in responding to H-ARS, the abundances of a selected profile of inflammatory proteins in serum were evaluated (Figure 2). These molecules were sorted into three groups. There were 14 pro-inflammatory (Figure 2A, IL-1a, IL-1b, IL-2, IL-3, IL-5, IL-6, IL-13, GM-CSF, IFN-gamma, KC, TNF-alpha, RANTES, IL-17a, and Eotaxin) and two anti-inflammatory markers (Figure 2B, IL-10 and G-CSF). Figure 2C depicted seven markers (IL-4, IL-9, MIP-1a, MCP, MIP-1b, IL-12p40, and IL-12p70) that can act as both pro- and anti-inflammatory factors.

Twelve of 14 pro-inflammatory markers in the irradiated saline mice showed a similar longitudinal monophasic trend. Their increasing abundances in serum reached a maximum at day 15 post-TBI, which was followed by a reduction in their concentration till the conclusion of this study at 30 d post-TBI (Figure 2A). On the other hand, the administration of JNJ-26366821 hindered the production of these pro-inflammatory agents.

Like the majority of pro-inflammatory proteins, two anti-inflammatory proteins, namely IL-10 and G-CSF, also showed a monophasic longitudinal trend among the irradiated saline mice (Figure 2B). JNJ-26366821 administration was able to arrest the surge.

Three of the 7 other inflammatory agents depicted in Figure 2C showed monophasic temporal patterns in the irradiated saline mice. Their concentration profiles peaked at 15 d post-TBI. In addition, the serum concentrations of MCP-1 and MIP-1b continuously increased in saline mice up to 30 d post-TBI. JNJ-26366821 administration was able to control the abundances of all these five (i.e., IL 4, IL-9, MIP-1a, MCP, and MIP-1b) proteins. The effect of JNJ-26366821 was sub-optimal in ameliorating IL12-p40 and IL12-p70.

### 3.2. Global Proteomics, Metabolomics and Lipidomics Profile Showed Omics-Specific Impacts of JNJ-26366821

**Figure 3:**
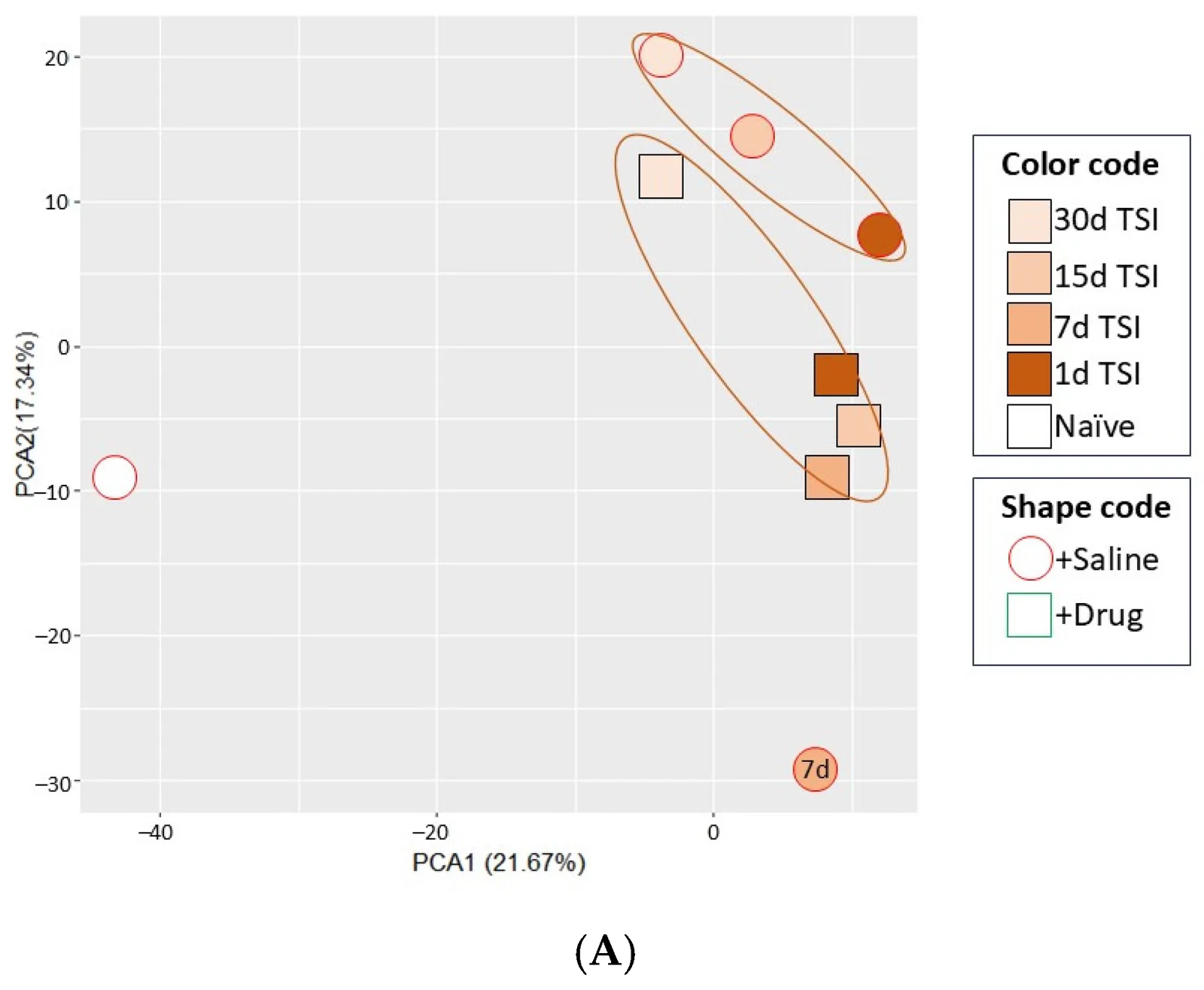

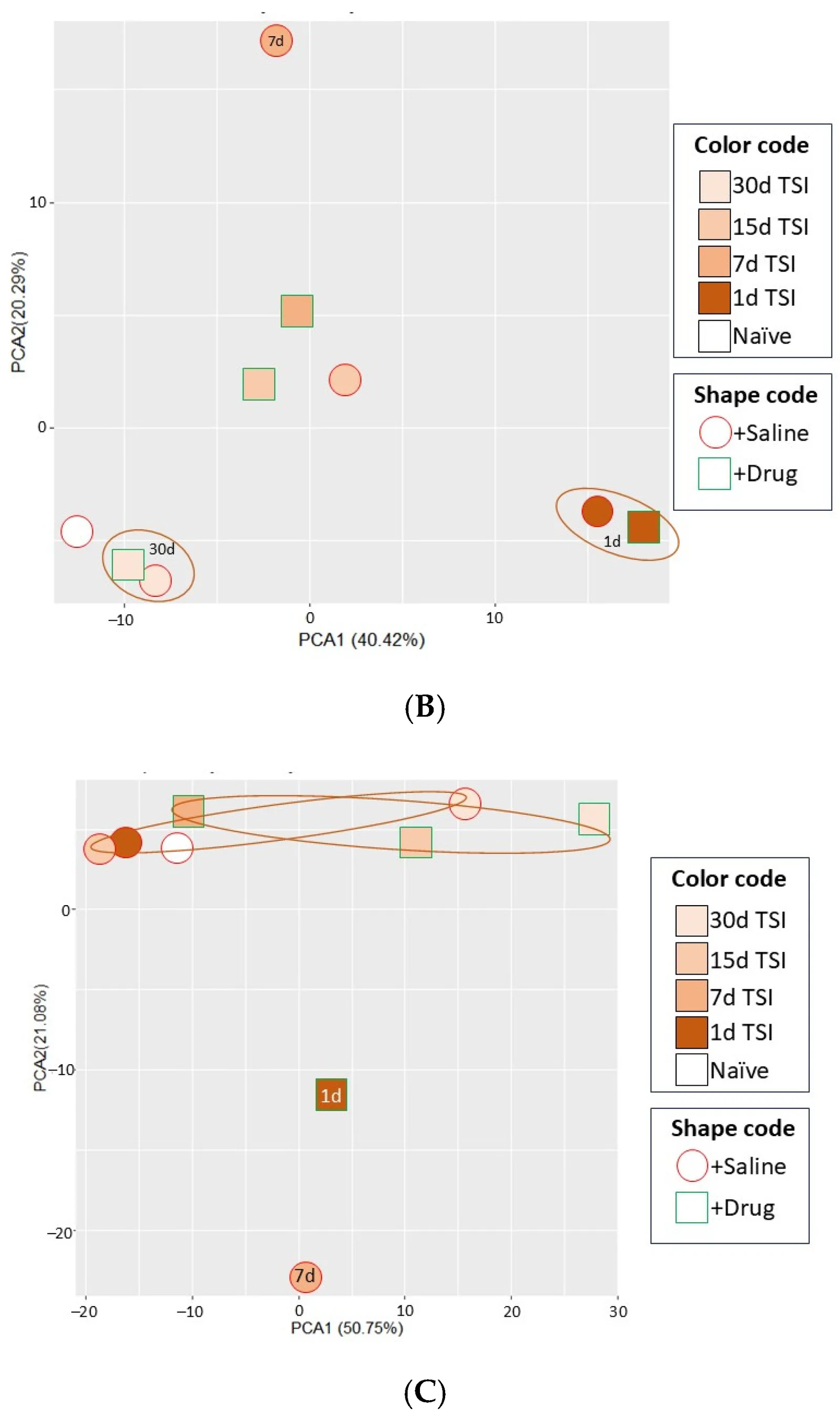
PCA plots related to the untargeted omics assay. The sample legends are on the right-hand side. The saline- and JNJ-26366821-treated samples are depicted by circle and square, respectively. The timepoints are color graded. (**A**) Proteomics assay. The post-irradiated saline samples except 7 d-TSI are clustered together and encircled. Likewise, all post-irradiated JNJ-26366821 samples are clustered together and encircled. (**B**) Metabolomics assay. The saline and JNJ-26366821 samples were clustered together according to the time since irradiation. (**C**) Lipidomics assay. The naïve and post-irradiated samples were largely clustered together, except 7 d-TSI saline and 1 d-TSI JNJ-26366821 samples, respectively.

Figure 3A displays the PCA plot of global proteomics data. PC1 and PC2 explained 21.67% and 17.34% of the total variance, respectively. A large separation along PC1 was observed between the naïve mice and the irradiated mice. Among the irradiated mice, the saline 7 d post-TBI mice clustered away from the rest along PC2. The bar and whisker plot of PC1 showed no major trend (Figure S2A), but there was significant temporal change in PC2. The values of PC2 related to 7 d post-TBI saline samples were significantly lower than those of 15 d and 30 d post-TBI saline samples (Figure S2B).

Figure 3B displays the PCA plot of global metabolomics data. PC1 and PC2 explained 40.42% and 20.29% of the total variance, respectively. The samples were primarily clustered by timepoints, but the saline 7 d post-TBI mice clustered away from the rest along PC2. Samples from both treatment groups, namely saline and JNJ-26366821 at 1 d post-TBI, clustered away from the naïve group along PC1; however, those at 30 d post-TBI clustered in proximity to the naïve mice. The bar and whisker plots of PC1 and PC2 showed significant temporal changes (Figure S2C,D).

Figure 3C displays the PCA plot of global lipidomics data. PC1 and PC2 explained 50.75% and 21.09% of the total variance, respectively. The samples were primarily clustered together, although the saline 7 d post-TBI mice and JNJ-26366821 1 d post-TBI mice clustered away from the rest along PC2. The bar and whisker plots of PC1 and PC2 showed no major trend (Figure S2E,F).

### 3.3. Differential Expression Analysis of Global Proteomics, Metabolomics and Lipidomics Profile

A *t*-test was conducted between saline-treated and JNJ-26366821-treated unirradiated mice to capture those molecular features in the heart that were altered due to the exclusive impact of JNJ-26366821. This comparative analysis yielded 30 proteins, 11 metabolites and 315 lipids that met the significance level of *p* < 0.05.

In parallel, a two-way ANOVA was conducted on the heart tissues extracted from the irradiated mice to understand the impacts of time since irradiation (TSI) and the JNJ-26366821 drug. Supplementary Table S1 lists the raw data, and Table S2 lists the differentially expressed proteins, metabolites and lipids. There were 27 proteins, four metabolites and two lipids that were differentially expressed by TSI at the significance threshold of *p* < 0.05. Likewise, there were 35 proteins, five metabolites and three lipids that were differentially expressed by TSI at the significance threshold of *p* < 0.1. There were three proteins, zero metabolites and zero lipids that were differentially expressed by drug treatment at the significance threshold of *p* < 0.05. Likewise, there were five proteins, zero metabolites and zero lipids that were differentially expressed by JNJ-26366821 drug treatment at the significance threshold of *p* < 0.1. Finally, there were 41 proteins, two metabolites and five lipids that were differentially expressed by the cumulative impacts of TSI and drug treatment at the significance threshold of *p* < 0.05. Likewise, there were 78 proteins, nine metabolites and 12 lipids that were differentially expressed by the cumulative impacts of TSI and drug treatment at the significance threshold of *p* < 0.1. Subsequent Venn analysis (Figure S3) curated those molecular features that were perturbed only by the cumulative impacts of TSI and drug treatment, and identified 76 proteins, nine metabolites and 12 lipids for functional analysis. Figure S4 shows volcano plots depicting the profile of the three omics components across treatment, TSI and treatment × TSI. Table 1 lists those proteins that were most expressed (log-fold change > |1.5|) at 1 d post-TBI.

### 3.4. Longitudinal Profile of Networks Perturbed by TBI and Therapeutic Intervention

Functional analysis of unirradiated mice with and without JNJ-26366821 treatment found seven protein networks that were perturbed by the exclusive effects of this drug (Table 2 (A)). In parallel, there were around 30 networks that were differentially perturbed by the therapeutic effects of JNJ-26366821 at different timepoints since irradiation (Table 2 (B)). In comparison, there was one network, namely cellular homeostasis that was common to Table 2 (A,B).

**Figure 4:**
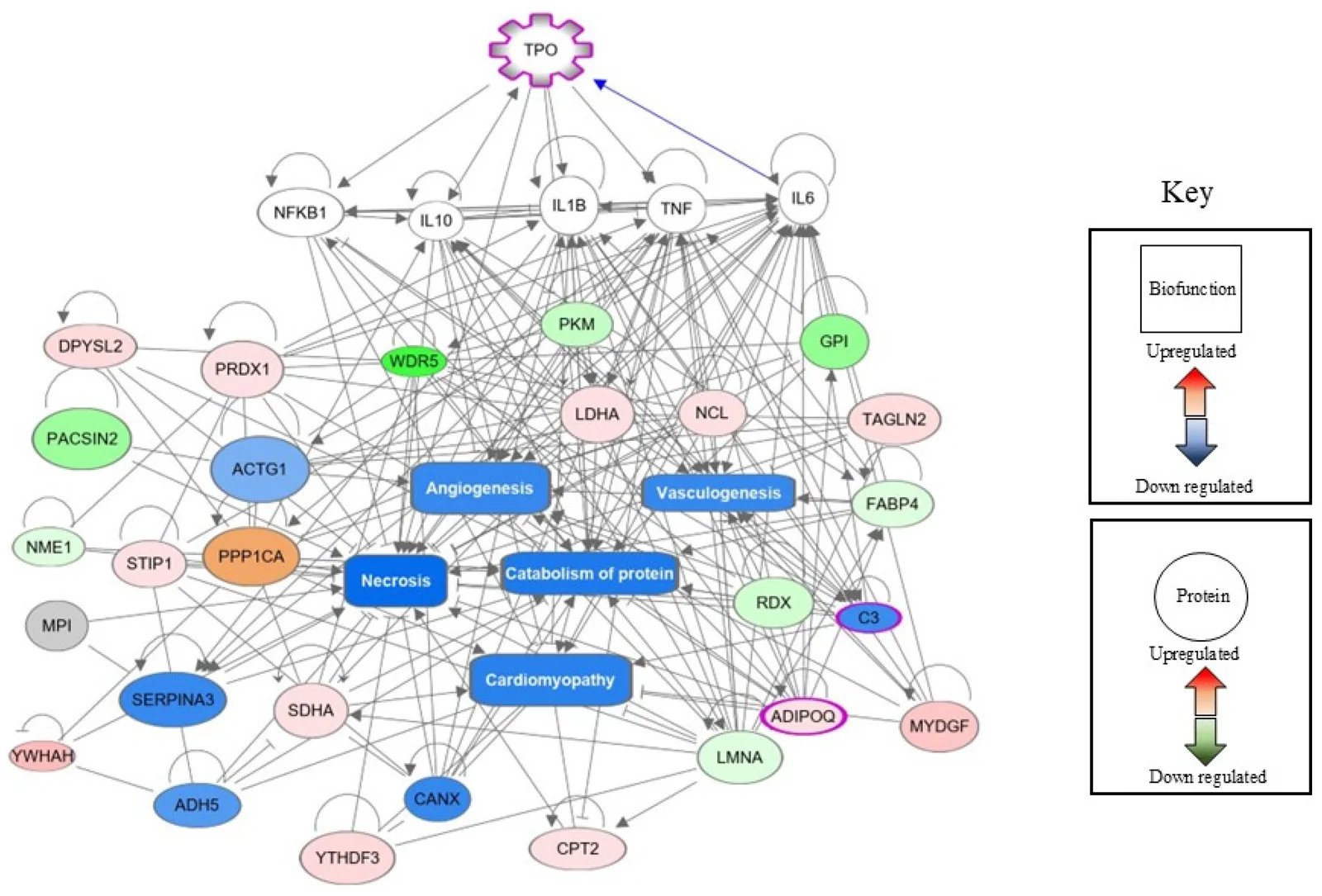
Functional network linked to cell death and cardiac health. The nodes are color coded based on their regulation status at one day (1 d) post-TBI. The edges connecting the nodes represent their relationship; arrow-headed: activated; blunt: association. The white nodes are not directly part of these networks but inserted as the intermediate between the network and TPO.

The protein network associated with necrosis emerged as one of the most highly enriched networks at the early and late timepoints, and this network was inhibited. Networks linked to cardiac health, such as angiogenesis, vasculogenesis and migration of epithelial tissues were significantly inhibited at early timepoints (Figure 4). There are 27 proteins in this cluster that were differentially perturbed by JNJ-26366821 in comparison to saline treatment. TPO is linked to this gene cluster through a group of immune-related proteins, namely IL-1b, IL-6, IL-10, TNFα and NFκb, and these proteins are depicted in Figure 4.

## 4. Discussion

The objective of the present work was to evaluate the efficacy of a novel TPOm, JNJ-26366821, as a mitigator of H-ARS-induced heart damage. The acute impact of H-ARS includes depletion of blood cell counts, which might induce multiple adverse implications including the impairment of cardiac functions [6]. The primary justification for selecting JNJ-26366821 in this study as a potential countermeasure resulted from the published study showing thrombopoietin’s capability to ameliorate thrombocytopenia; and its history of stabilizing cardiac diseases [6].

The current study was modeled based on previous work that had reported several benefits of JNJ-26366821 as a mitigating intervening agent [12]. Kumar et al. have demonstrated a >70% increased rate of survival from a lethal dose of 9.35 Gy TBI with a single dose of JNJ-26366821 at 0.3 mg/kg administered 24 h post-TBI. The 30-day long survival curves were generated using CD2F1 mice and substantiated by C57BL/6 mice. The dose reduction factor analysis in C57BL/6 mice showed minimal (~5%) mortality with intervention with JNJ-26366821, whereas there was an >80% chance of mortality in mice administered saline. At a lethal dose of 8.5 Gy TBI, the chance of mortality increased to ~50% in the C57BL/6 mouse model [12]. The impact of JNJ-26366821 included recovery of femoral bone marrow as estimated by colony forming unit (CFU) and megakaryocytes; H&E-stained slides of sternal bone marrow sections showed restored bone marrow cellularity after the therapeutic intervention of JNJ-26366821. In general, these blood cells were significantly recovered after an initial lag, with recovery beginning 7 d–10 d post-7.0 Gy sublethal dose countered by the post-exposure intervention of JNJ-26366821 [12].

A global proteomic, metabolomics and lipidomics study suggested that JNJ-26366821 predisposed the system by activating several biological networks that can potentially protect the host from radiotoxicity. For instance, the administration of JNJ-26366821 to unirradiated mice promoted cellular homeostasis, cell viability and gene activation networks, which were upheld after its therapeutic intervention, and potentially induced some pro-survival mechanisms. H-ARS acutely triggers cell death [32,33], but JNJ-26366821 inhibited the protein networks linked to necrosis immediately after the therapeutic intervention. The differentiation of adipocytes and the development of epithelial tissue were activated 7 d and 15 d post-TBI, respectively. Past studies showed that even clinically relevant radiation doses can acutely and persistently damage the adipocytes and epithelial tissue formation [34]. Furthermore, the administration of JNJ-26366821 to naïve mice upregulated the protein–metabolite networks linked to the scavenging of free radicals, which would be primarily beneficial since the generation of free radicals and peroxides is a common indirect effect of radiation that causes severe damage to the peripheral tissues [32].

The assay of the blood immune profile of inflammatory cytokines suggested that JNJ-26366821 potentially bridged the innate immune system to its adaptive arm but arrested the inflammatory surge caused by lethal radiation. This post-exposure intervention by the drug potentially primed the T cells by maintaining the regulation of IFNγ, IL-1a, IL1RP and IL-1b at two-fold higher than that of the unirradiated baseline. On the same note, JNJ-26366821 orchestrated a controlled activation of granulocyte-macrophage colony-stimulating factor (GM-CSF) and granulocyte colony-stimulating factor (G-CSF). An uncontrolled propagation of GM-CSF and G-CSF can potentially promote tumorigenesis and an inflammation surge, which could outweigh their beneficial effects, such as ameliorating neutropenia [35,36]. Increased blood cell counts and recovery of cellularity of sternal bone marrow on 30 d post-8 Gy TBI [12] supported the tenet that the controlled regulation of GM-CSF and G-CSF by JNJ-26366821 potentially helped in blood cell recovery. In concurrence, JNJ-26366821 potentially promoted adaptive immunity by upregulating the networks linked to endocytosis and molecular transport starting from 7 d post-TBI. Past studies showed that such methods, namely co-promoting adaptive and innate immunity, could be instrumental in tumor rejection [37].

A comprehensive longitudinal study of the functional networks revealed that the acute impact of JNJ-26366821 potentially failed to ameliorate cardiac damage caused by lethal H-ARS, as the networks linked to angiogenesis, vasculogenesis, migration of endothelial cells, which are typical markers of healthy cardiac functions [38,39] were inhibited following 1 d post-TBI intervention. Furthermore, at that stage, some of the key protein markers of pro-cardiac health, such as RAD23A [40] and TTN [41] were inhibited, and PPP1CA [42], which is known to be highly abundant at the end stage of heart failure, was upregulated. Over the next seven days, as the blood cells recovered following JNJ-26366821 treatment [12], pro-inflammatory proteins started operating in restrained modes and the cachexia signaling pathway [43] became inhibited, which potentially arrested muscle proteolysis and regenerative functions. In concurrence, JNJ-26366821 activated several networks linked to cellular maintenance, such as organization of cytoplasm, and synthesis and metabolism of protein. By 30 d post-TBI, additional biofunctions like glycolysis, and the metabolism and uptake of several macromolecules became activated to support the cellular maintenance. This longitudinal study underscored the chronic impacts of JNJ-26366821 to promote healthy cardiac functions.

## 5. Conclusions, Limitations and Future Work

This study demonstrates that a single subcutaneous dose of the novel PEGylated TPOm, JNJ-26366821, administered 24 h post-exposure, acts as an effective mitigator against radiation-induced tissue damage in a lethal (8.0 Gy) murine model of H-ARS. By integrating serum cytokine profiling with a multi-omics approach (proteomics, metabolomics, and lipidomics), this investigation provides a systemic, timeline-driven map of the drug’s therapeutic efficacy within the cardiovascular system.

The comprehensive findings of this research suggested several critical insights. Nevertheless, it is important to note that these conclusions were primarily drawn from serum cytokines, cardiac omics data and molecular network inference. The current study did not include direct functional or histological validation of cardiac injury or recovery. JNJ-26366821 successfully restrained the radiation-induced hyper-inflammatory cytokine surge by day 7 post-TBI. It modulated the expression of key pro-inflammatory mediators (e.g., IL-6, TNF-α, IL-1β) and orchestrated a balanced, controlled activation of hematopoietic growth factors (G-CSF and GM-CSF), supporting safe hematopoietic recovery without sustaining systemic toxicity. At the acute stage (day 1 post-TBI), the countermeasure immediately suppressed cellular necrosis networks and prioritized cellular survival pathways, potentially countering the initial wave of radiation-induced cell death. While early post-intervention networks showed a transient inhibition of angiogenesis and endothelial migration, a therapeutic pivot occurred on day 7. JNJ-26366821 mobilized tissue-specific molecular networks to drive cellular maintenance, downregulate cachexia signaling, and prevent radiation-induced muscle wasting. Between days 15 and 30 post-exposure, the drug actively upregulated vital cardioprotective mechanisms, including protein synthesis, glycolysis, and macromolecular transport, effectively restoring myocardial energy homeostasis.

While these findings suggest a strong molecular foundation for JNJ-26366821, certain limitations of the current study should be noted. The evaluation was restricted to a single mouse strain and a single sex (males only), despite independent studies from our group and others demonstrating significant sex bias in radiation responses. Additionally, expanding the sample size would further strengthen these findings. However, previous validation across different strains, combined with the multi-omics integration employed in the present study, provides statistical assurance in the current results. We acknowledge that untargeted metabolomics and proteomics assays carry an inherent risk of false positive and negative results. To address this, we implemented a two-step data curation process. First, we performed a two-way ANOVA to identify spectral features significantly perturbed by treatment, TSI, or their interaction. Second, we used a Venn analysis to exclude features that were affected exclusively by JNJ-26366821. This systematic down-selection method was chosen to maximize the validity of the insights drawn from our sub-optimal sample size. Finally, our approach emphasized mechanistic discovery through comprehensive network analysis rather than the identification of single molecular markers.

Moving forward, future studies must incorporate female cohorts to address the known sex difference, alongside cross-strain validations. Furthermore, incorporating functional cardiac assays—such as echocardiography—and formal myocardial histopathology will be essential to definitively link these multi-omic network changes to physical tissue recovery. In summary, despite these limitations, the present data strongly supports the potential of JNJ-26366821 as a safe, highly viable medical countermeasure against acute radiation syndrome. As a stable, non-immunogenic peptide requiring only a single subcutaneous dose 24 h post-exposure, it offers a streamlined clinical translation path. Ultimately, JNJ-26366821 stands out as a promising candidate that not only enhances immediate systemic survival following a radiological emergency but also mitigates the late-stage, life-limiting cardiotoxic complications traditionally associated with severe radiation exposure.

## Figures and Tables

**Table 1:** List of proteins showing maximum differential regulation (LogFC > |1.5|) immediately after the drug administration. Longitudinal trends of these proteins’ expression values are shown here.

Protein ID	Protein Name	Log_2_FC 1 d → 30 d TSI	Functions
IL1RP	Interleukin 1 receptor accessory protein	1.68 → ~0	Activates IL1 family, which was surged by irradiation and therapeutic administration of JNJ-26366821 restrained the regulations.
Lz1C	Leucine zipper and CTNNBIP1 domain containing	1.56 → ~0	A potential tumor suppressor
HDHD2	Haloacid dehalogenase like hydrolase domain containing 2	2.66 → 0.66	Regulates enzyme binding activities
TTN	Titin	−1.7 → ~0	Putative radiation biomarker, an essential structural entity of heart muscle.
RAD23A	UV excision repair protein RAD23 homolog A	−1.57 → 1.18	Involved in nucleotide excision repair (NER)

**Table 2:** List of biofunctions. The networks with z score > |1.5| and number of molecules ≥5 are listed here. (**A**) These biofunctions were significantly enriched by the group of proteins, metabolites and lipids that were differentially expressed between unirradiated mice with and without JNJ-26366821 treatment. (**B**) These biofunctions were significantly enriched by the group of proteins, metabolites and lipids that were differentially expressed between the irradiated mice with therapeutic treatment of JNJ-26366821 and without treatment (Saline). These networks are listed based on the days since irradiation.

(**A**)			
**Diseases or Functions Annotation**		**Activation z-Score**	**Molecules**
Activation of gene		1.54	6
Cellular homeostasis		1.55	7
Free Radical Scavenging		1.56	5
Assembly of organelle		1.75	6
Transcription of RNA		1.89	8
Transcription of DNA		2.01	8
Cell viability		2.46	11
(**B**)			
	**Diseases or Functions Annotation**	**Z-score**	**of Molecules**
Day 1	Necrosis	−2.25	24
	Vasculogenesis	−2.01	8
	Migration of endothelial cells	−1.90	6
	Angiogenesis	−1.73	9
	Cellular homeostasis	1.82	12
Day 7	Differentiation of adipocytes	1.97	5
	Cachexia Signaling Pathway	−2.00	5
	Endocytosis	1.68	7
	Secretion of protein	1.67	11
	Transport of molecule	2.05	21
Day 15	Development of epithelial tissue	1.71	7
	Differentiation of adipocytes	1.97	4
	Neutrophil degranulation	−2.24	7
	Organization of cytoplasm	1.63	11
	Organization of cytoskeleton	1.63	10
	Microtubule dynamics	1.64	7
	Metabolism of protein	1.65	37
	Synthesis of protein	1.78	33
	Sensitivity of cells	1.93	13
	Processing of RNA	−1.95	8
Day 30	Necrosis	−3.19	24
	Metabolism of macromolecule	1.69	38
	Synthesis of protein	1.69	33
	Neutrophil degranulation	1.89	7
	Uptake of monosaccharide	1.94	4
	Glycolysis of cells	2.39	6
	Translocation of protein	1.56	7
	Transport of molecule	1.67	21
	Secretion of protein	1.82	11
	Migration of cells	2.00	26

## Data Availability

The original contributions presented in this study are included in the article/Supplementary Material. Further inquiries can be directed to the corresponding authors.

## Supplementary Materials

The following supporting information can be downloaded at: https://www.mdpi.com/article/10.3390/biom16091271/s1, Figure S1. QC plots. (A) Internal Standards of metabolomics assay. (B) Internal Standards of lipidomics assay. (C) Minimal injection order effects for metabolomics assay. (D) Minimal injection order effects for lipidomics assay. All these data suggested that the internal standards were stable across batches, ensuring technical consistency and the minimal injection order effects showed no significant drift during runs. Figure S2. Bar and whisker plots of PC1 and PC2 values. * *p* < 0.05, ** *p* < 0.01, *** *p* < 0.001. (A) PC1 of proteomics data, (B) PC2 of proteomics data, (C) PC1 of metabolomics data, (D) PC2 of metabolomics data, (E) PC1 of lipidomics data, (F) PC2 of lipidomics data. Figure S3. Venn diagrams demonstrating comparison of molecules altered due to the cumulative impact of JNJ-26366821 drug and 8.0 Gy TBI (Drug × 8.0 Gy TBI) and the sole impact of JNJ-26366821 drug. (A) Proteins, (B) Metabolites, (C) Lipids. Only those molecules which were unique to the cumulative impact of JNJ-26366821 drug and 8.0 Gy TBI were used for functional analysis. Figure S4. Volcano plots showing the molecular profile across treatment, TSI and treatment × TSI. (A) Protein, (B) Metabolite, (C) Lipids. Table S1. (A) Protein data, machine normalized and before differential expression analysis. (B) Lipid data, machine normalized and before differential expression analysis. (C) Metabolite data, machine normalized and before differential expression analysis. Table S2. (A) ANOVA results of proteins. (B) ANOVA results of metabolites. (C) ANOVA results of lipids. (D) Proteomics, *t*-test between naïve sham vs. drug. (E) Metabolomics, *t*-test between naïve sham vs. drug. (F) Lipidomics, *t*-test between naïve sham vs. drug.

## Abbreviations

AFRRI	Armed Forces Radiobiology Research Institute
ARS	Acute radiation syndrome
CAD	Coronary artery disease
ESR	Electron Spin Resonance
IGF	Insulin growth factor
IL	Interleukin
miRNAs	microRNAs
PEG	Polyethylene glycol
TBI	Total body irradiation
TGFβ1	Transforming growth factor beta 1
TPO	Thrombopoietin
TPOm	Thrombopoietin mimetic
VCAM	Vascular cellular adhesion molecule
